# Laser light retinopathy

**Published:** 2019

**Authors:** Carmen Alba-Linero, Carlos Rocha de Lossada, Marina Rodríguez Calvo de Mora, Ramirez Nieves de las Rivas, Ayala Carlos Hernando

**Affiliations:** * Ophthalmology Department, Hospital Civil, Hospital Regional of Malaga, Malaga, Spain; ** Faculty of Medicine, University of Malaga (UMA), Malaga, Spain

**Keywords:** laser light, macular hemorrhage, retinopathy, optical coherence tomography

## Abstract

**Objective:** To report a clinical case of macular retinopathy after laser light exposition.

**Methods:** We described a case of reversible maculopathy in a 29-year-old woman. Retinography and Optical Coherence Tomography were performed.

**Discussion:** Retinopathy due to laser light is an increasingly frequent pathology because of its improper use and the massive sale not regulated by the Internet. These lesions can vary from mild disruptions in the pigmentary epithelium to retinal hemorrhages, retinal ruptures, or macular holes. The depth of the lesion and the involvement of the photoreceptors layer will confer a worse visual prognosis.

**Conclusion:** A correct control of the sale and consumption of these devices are necessary to suppress this completely avoidable pathology.

## Introduction

Laser light retinopathy is an increasingly common pathology due to the spread of Internet sales and less control of the safety of laser light devices. Laser lights are often mis-labeled and mis-marketed as toys and they are sold to all kinds of consumers, even children. However, even so-called safe lower-power lasers may produce retinal damage and permanent visual loss [**1**]. 

The human eye suffers damage as a result of ionization, thermal and photochemical mechanisms when it is exposed to laser light of more than 500 mW of power and between 400 and 1400 nm of wavelength [**2**].

There is a classification of laser devices according to their power. They are classified from I to IV, those labeled as category IIIb and IV being unfit for consumption [**3**]. 

The aim of this case report was to describe a laser light retinopathy of a young woman on a dance floor. 

## Case report

A 29-year-old woman without any previous ocular or systemic antecedent came to the emergency department of ophthalmology for a central scotoma of the right eye (OD) from the night before, when her eye was pointed with a laser on a dance floor. She referred a laser light exposition of 10 seconds. After the exposition, she felt a permanent central scotoma.

Visual acuity of the OD was 20/ 200 (decimal scale, Snellen chart) with a defect in the central visual field. Anterior pole exam by slit-lamp was anodyne. 

**Fig. 1:**
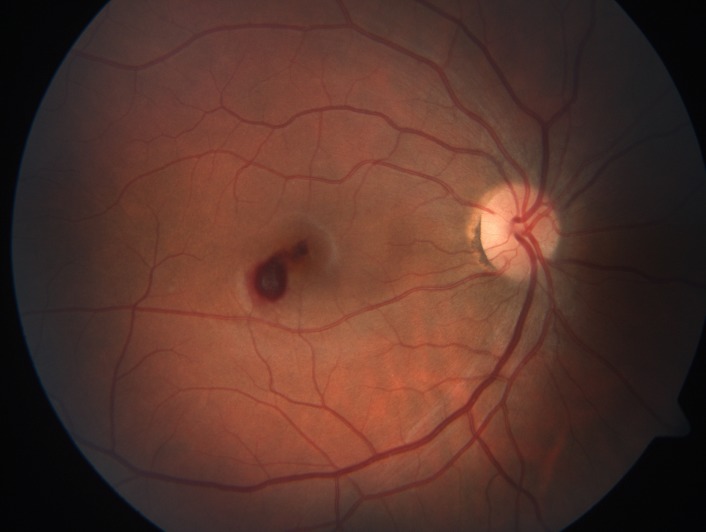
Right eye retinography showing a central epiretinal and intraretinal hemorrhage

On fundoscopic examination, an elevated hemorrhagic lesion was seen in the macular area (**Fig. 1**).

**Fig. 2:**
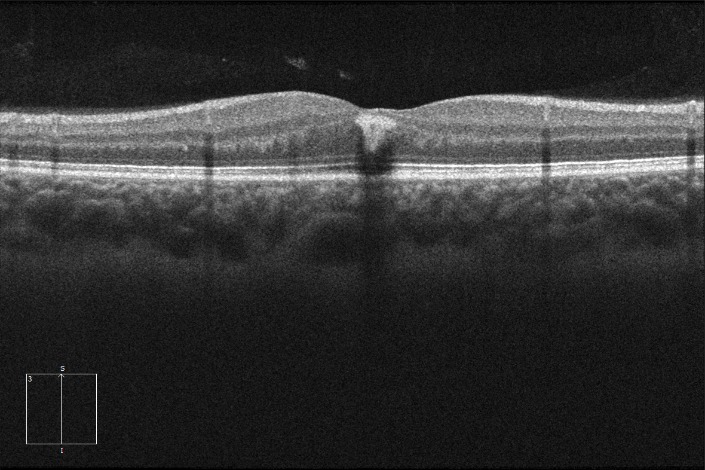
Optical Coherence Tomography revealing an inner retinal hyperreflective encapsulated lesion in foveal zone

An OCT (optical coherence tomography) was performed, which showed a hyper-reflective lesion compatible with parafoveal hemorrhage encapsulated in the inner layers of the retina (**Fig. 2**).

**Left eye examination (OS) was normal.**

We decided to do an expectant management with rest and observation of the patient.

One month later the patient had a total recovery of visual acuity in OD (20/ 20) with a total resolution of the retinal hemorrhage.

## Discussion

Laser lesions in the posterior pole can range from minimal hypertrophy of the pigment epithelium to the appearance of central serous chorioretinopathy, hemorrhage, choroidal neovascularization, or macular hole [**4**].

Bilateral retinopathy has been reported previously and may be more likely to occur in cases involving mirrors or beam splitters or in patients with alternating fixation [**5**]. Although the use of low powered lasers is widespread, fearful children may report symptoms only if bilateral visual impairment has occurred [**6**].

The reversibility of the same is related to the consequent disruption of the photoreceptors layer. In the case we presented, the affection was in the inner retinal layer, so visual and anatomic recovery was satisfactory. 

An adequate control of the sale to the public of high-power laser devices is necessary to avoid these pathologies either by direct exposure or by the glare of the light [**7**]. A greater regulation of Internet sale, age restriction of use, and education are essential to avoid the damage of these dangerous devices. 

**Conflict of interest**

There is no conflict of interest. 

All authors agree with the publication of this manuscript. 

This clinical investigation was approved by the Ethics Committee of the Hospital Regional of Malaga and adheres to the principles of the Declaration of Helsinki.

The patient was informed and signed the informed consent to participate in this study. 
